# High HIV-1 prevalence, risk behaviours, and willingness to participate in HIV vaccine trials in fishing communities on Lake Victoria, Uganda

**DOI:** 10.7448/IAS.16.1.18621

**Published:** 2013-07-22

**Authors:** Noah Kiwanuka, Ali Ssetaala, Juliet Mpendo, Matthias Wambuzi, Annet Nanvubya, Simon Sigirenda, Annet Nalutaaya, Paul Kato, Leslie Nielsen, Pontiano Kaleebu, Josephine Nalusiba, Nelson K Sewankambo

**Affiliations:** 1 Department of Epidemiology and Biostatistics, School of Public Health, Makerere University College of Health Sciences, Kampala, Uganda; 2 UVRI-IAVI HIV Vaccine Program, Entebbe, Uganda; 3 International AIDS Vaccine Initiative (IAVI), NY, USA; 4 Laboratory and Basic Sciences department, Uganda Virus Research Insitute, Entebbe, Uganda; 5 Clinical Epidemiology Unit, School of Medicine, Makerere University College of Health Sciences, Kampala, Uganda

**Keywords:** HIV-1, willingness to participate in HIV vaccine trials, fishing communities, Uganda

## Abstract

**Introduction:**

HIV epidemics in sub-Saharan Africa are generalized, but high-risk subgroups exist within these epidemics. A recent study among fisher-folk communities (FFC) in Uganda showed high HIV prevalence (28.8%) and incidence (4.9/100 person-years). However, those findings may not reflect population-wide HIV rates in FFC since the study population was selected for high-risk behaviour.

**Methods:**

Between September 2011 and March 2013, we conducted a community-based cohort study to determine the population representative HIV rates and willingness to participate (WTP) in hypothetical vaccine trials among FFC, Uganda. At baseline (September 2011–January 2012), a household enumeration census was done in eight fishing communities (one lakeshore and seven islands), after which a random sample of 2200 participants aged 18–49 years was selected from 5360 individuals. Interviewer-administered questionnaire data were collected on HIV risk behaviours and WTP, and venous blood was collected for HIV testing using rapid HIV tests with enzyme-linked immunosorbent assay (EIA) confirmation. Adjusted prevalence proportion ratios (adj.PPRs) of HIV prevalence were determined using log-binomial regression models.

**Results:**

Overall baseline HIV prevalence was 26.7% and was higher in women than men (32.6% vs. 20.8%, *p*<0.0001). Prevalence was lower among fishermen (22.4%) than housewives (32.1%), farmers (33.1%) and bar/lodge/restaurant workers (37%). The adj.PPR of HIV was higher among women than men (adj.PPR =1.50, 95%; 1.20, 1.87) and participants aged 30–39 years (adj.PPR=1.40, 95%; 1.10, 1.79) and 40–49 years (adj.PPR=1.41, 95%; 1.04, 1.92) compared to those aged 18–24 years. Other factors associated with HIV prevalence included low education, previous marriage, polygamous marriage, alcohol and marijuana use before sex. WTP in hypothetical vaccine trials was 89.3% and was higher in men than women (91.2% vs. 87.3%, *p*=0.004) and among island communities compared to lakeshore ones (90.4% vs. 85.8%, *p*=0.004).

**Conclusions:**

The HIV prevalence in the general fisher-folk population in Uganda is similar to that observed in the “high-risk” fisher folk. FFC have very high levels of willingness to participate in future HIV vaccine trials.

## Introduction

Globally, the HIV pandemic is most severe in East and Southern African countries, where the regional epidemics are generalized and stable or declining [1]. In generalized HIV epidemics, transmission of the virus is not primarily occurring in or confined to groups at high risk of infection but rather is spread across the general population where at least 1% is infected [1]. Groups at high risk of HIV infection can co-exist within generalized epidemics and may be masked by estimates made from the general population [2].

Emerging evidence in Uganda suggests that fisher-folk communities (FFC) are one of the groups at high risk of HIV infection within a mature and generalized epidemic [2–4] but robust epidemiological data particularly from representative populations are still scanty. A recent study in FFC found a prevalence of 28.8% and an incidence rate of 4.9/100 person-years [2, 4]; figures are about four to five times higher than the national averages [5]. These findings may not reflect population-wide HIV rates in FFC since the study comprised only persons who were identified as being at high risk of HIV infection based on the recent history (three months) of number of sexual partners, unprotected sexual intercourse with new partners and symptoms of sexually transmitted infections at screening. More studies from representative FFC populations are needed to determine population-wide HIV rates, risk factors associated with infection and patterns of transmission within these communities. If fishing communities are characterized as another group at high risk for HIV, specific prevention and control efforts targeting these communities will be urgently required to mitigate the spread of HIV within FFC and to the general population. Furthermore, these populations may be appropriate for assessing the efficacy and broader impact of interventions including trials of combination prevention and HIV vaccines. We conducted a community-based cohort study in a randomly selected population representative sample in eight fishing communities around Lake Victoria, Uganda, to determine whether the population-based HIV rates and associated factors, are similar to those observed in persons selected for high-risk behaviours, and to evaluate the level of willingness to participate (WTP) in future HIV vaccine trials. In this article, we report baseline findings on HIV-1 prevalence and its associated factors, and WTP.

## Methods

### Study sites

Eight FFC (one lakeshore and seven islands) in three Uganda districts of Wakiso (four communities), Mukono (two communities) and Kalangala (two communities) were purposively selected based on zero participation in previous HIV epidemiological studies, geographical representation and size (relatively large communities with an estimated adult population size of ~1000). Relatively large communities were preferred because of the potential for expansion in case of large studies in the future.

### Sample size estimation

We assumed that the HIV prevalence and incidence in the general FFC population were approximately 20–30% lower than those observed among the high-risk FFC persons (prevalence=28.8% and incidence 4.9/100 person-years at risk-py), respectively [2]. Using a prevalence of 20% (which would give a bigger sample size), an alpha (*α*) of 0.05, a precision of 5% and a non-response rate of 10%, we obtained a total sample size of 2200 participants. That sample size would enable estimation of a baseline HIV prevalence of 20% or higher and an incidence of 3.5/100 person-year or higher at 18 months follow-up with a precision of around ±1.2.

### Study population and data collection

Between September 2011 and March 2013, we conducted a community-based cohort study to determine the population representative of HIV rates and WTP in future vaccine trials among FFC, Uganda. At baseline (September 2011–January 2012), a household enumeration census was conducted in eight fishing communities. To obtain a population representative sample, all households in the selected communities were mapped and each was assigned a unique household number. Within a household, all adults and children were listed and each member was assigned a unique identification member number. A detailed enumeration questionnaire was administered to the head of the household or a proxy and census data were collected on each household member with regard to age, sex, relationship to household head, duration of residence in community and marital status. A complete census list of all persons aged 18–49 years (3269 in total) was generated from which 2200 participants were selected through proportionate random sampling using Stata^®^ 11 (StataCorp, College Station, TX, USA) software. Each community's proportionate contribution to the study population of 2200 was determined by (2200/3269)*y_*i*_, where *y*_*i*_ was the total number of persons aged 18–49 years in a given community. Selected participants who provided written informed consent were enrolled and interviewed in privacy by same-sex interviewers using a semi-structured questionnaire. At interview, data were obtained on socio-demographic characteristics, HIV risk sexual behaviours, number of partners, condom use, alcohol and illicit drug use. Venous blood was taken for HIV-1 serology and participants received pre- and post-test counselling from certified HIV counsellors. Participants who opted to receive their HIV results got them and those infected with HIV were referred to HIV/AIDS care centres for further management. Counsellors encouraged participants to share their HIV results with their sexual partners since involuntary disclosure of HIV results to partners is not allowed under the Ugandan Ministry of Health AIDS Control Program policy on HIV testing [6]. Data were also collected from all participants (regardless of HIV status) on knowledge of HIV vaccines and WTP in future HIV vaccine studies. Participants were asked if they were aware of efforts to develop and test candidates’ vaccine for HIV prevention and if they were willing to be a study participant in a trial of an HIV preventive vaccine in case one became available for testing in their community. The questions on WTP were hypothetical since there was no actual HIV vaccine trial taking place in the communities during the study. Ethical reviews and approval were obtained from Uganda Virus Research Institute's Science and Ethics Committee (FWA number 00001354, expires on February 2013) and the Uganda National Council for Science and Technology (FWA number 00001293).

### Laboratory testing

Rapid HIV tests were performed in the community by certified laboratory technologists as per the Uganda National HIV Testing Algorithm. All blood samples were first tested on Determine^®^ HIV assay (ABBOTT Laboratories, Diagnostic division, Chicago, IL, USA), and if negative, results were reported as negative. All Determine^®^-positive samples were further tested using HIV 1/2 Stat-Pak^®^ assay (Chembio Diagnostic Systems, Inc. NY, USA), and if also positive, results were reported as positive. But if negative on Stat-Pak^®^, Uni-Gold™ Recombigen^®^ HIV test (Trinity Biotech, USA) was used as a tiebreaker. All positive rapid results were confirmed using two sequential enzyme-linked immunosorbent assay (EIA) tests: Vironostika (HIV Uni-Form II plus 0 microelisa system, Biomerieux, Boxtel, The Netherlands); and Murex HIV-1.2.0 (Murex, Biotech Limited, Dartford, UK). For discordant EIA results, Stat-Pak^®^ was used as a tiebreaker.

### Data management and statistical analysis

Field-based data editors checked completed data collection forms for accuracy, consistency, and completeness soon after collection and errors were corrected in the field. Data were double entered using EPI-DATA entry screens with range, consistency and logic checks. The two entries were compared and any discordances found were rectified by reference to source documents. Participants’ baseline characteristics were summarized and compared using *t*-tests for continuous variables and Chi-square and Fisher Exact tests for categorical variables. Given the high prevalence of the outcome (HIV prevalence=26.7%), we used log-binomial regression models to estimate unadjusted and adjusted prevalence proportion ratios (PPRs) and corresponding 95% CIs of factors associated with HIV prevalence. In order to account for potential correlation at household level (where more than one participant from a given household was selected), we used an empirical variance estimator to determine robust standard errors associated with PPRs. When the proportion of the outcome is greater than 10%, odds ratios give biased estimates of prevalence ratios, hence the choice of log-binomial regression over logistic regression [7]. Covariates were selected for inclusion in multivariable models based on biological plausibility and a bivariate statistical significance at an *α* of <0.15. An exploratory logical model building method was used and the final model was adjusted for age, sex, education, religion, marital status, alcohol consumption in the past three months, alcohol use before sex and the use of marijuana. Statistical analyses were performed using Stata^®^ 11 (StataCorp, College Station, TX, USA) software.

## Results

Of the 2200 selected participants, 2191 (99.6%) provided written informed consent and were enrolled in the study. Of these, 419 (19.1%) came from Kiimi, 278 (12.7%) from Namisoke, 117 (5.3%) from Myende, 300 (13.7%) from Zinga, 247 (11.3%) from Kavenyanja, 381 (17.4%) from Kigungu and 170 (7.8%) from Makusa. There were no differences with regard to age (18–49 years, *p*=0.64) and gender (*p*=0.52) between enrolled participants and overall population reflected in the community census (not shown). As shown in Table 1, 50.5% of the study population were men, the overall mean (SD) and median (IQR) age were 29.7 (7.6) and 29 (24–35) years, respectively. Eighty-seven per cent (87.4%) of all the participants were aged 18–39 years. The majority (59.1%) attained primary education and 47.3% were engaged in fishing activities including fishing, fish selling and processing, and boat making, and nearly 20% were engaged in businesses including bars, lodges and restaurants. Forty-two per cent reported being in monogamous marriages, 19.3% in polygamous marriages, and 23.1% had previously been married but were not married at the time of interview. Fifty-six per cent of the participants had spent less than five years in the fishing communities, 52.9% consumed alcohol in past three months, 43.4% reported alcohol use before sex in the same time frame, and 13.8% reported use of illicit drugs such as marijuana. Age, alcohol consumption, use of illicit drugs and fishing activities were significantly higher among men than women. Among the women, 93.5% (1015/1085) had ever been pregnant and 9.3% reported being pregnant at the time of the study. The mean (SD) and median (IQR) age at first pregnancy was 18 (7.9) years and 17 (15–19) years, respectively and median (IQR) number of live births per woman was 3 (2–5). Overall, 40.1% (806/2008) reported condom use in the past 12 months of whom 24.6% (198/806) reported consistent condom use; the latter did not differ by gender (*p*=0.28).

**Table 1:** Baseline socio-demographic characteristics of the study population by gender (*n*=2191)

	All	Male	Female	
		
	2191 (99.5%)	1106 (50.5%)	1085 (49.5%)	*p*
Age at enrolment (years)				
Mean (SD)	29.7 (7.6)	30.8 (7.6)	28.7 (7.5)	<0.0001
Median (IQR)	29.0 (24–35)	30.0 (25–36)	27.0 (23–33)	<0.0001
18–24	616 (28.1%)	261 (23.6%)	355 (32.7%)	
25–29	566 (25.8%)	278 (25.1%)	288 (26.5%)	
30–39	733 (33.5%)	407 (36.8%)	326 (30.0%)	
40–49	276 (12.6%)	160 (14.5%)	116 (10.7%)	<0.0001
Age of sexual debut (years)				
Mean (SD)	17.3 (8.3)	18.0 (8.4)	16.5 (8.3)	<0.0001
Median (IQR)	16.0 (15–18)	17.0 (15–19)	16.0 (14–17)	<0.0001
Highest education level*				
None	186 (8.5%)	82 (7.4%)	104 (9.6%)	
Primary	1294 (59.1%)	652 (59.1%)	642 (59.2%)	
Post-primary	708 (32.4%)	369 (33.4%)	339 (31.2%)	0.149
Religion				
Roman Catholic	890 (40.6%)	450 (40.7%)	440 (40.5%)	
Protestant/Anglican	600 (27.4%)	320 (28.9%)	280 (25.8%)	
Islam	421 (19.2%)	219 (19.8%)	202 (18.6%)	
Pentecostal	197 (9.0%)	78 (7.0%)	119 (11.0%)	
Other¶	83 (3.8%)	39 (3.5%)	44 (4.1%)	0.017
Ethnicity/tribe				
Non-Muganda	1197 (54.6%)	646 (54.0%)	551 (46.0%)	
Muganda	994 (45.4%)	460 (46.3%)	534 (53.7%)	<0.0001
Occupation				
Fishing/fishing-related	1038 (47.4%)	817 (73.9%)	221 (20.4%)	
Trade/business	223 (10.2%)	54 (4.9%)	169 (15.6%)	
Bar/lodge/restaurant	257 (11.7%)	15 (1.4%)	242 (22.3%)	
Farming	130 (5.9%)	60 (5.4%)	70 (6.4%)	
Others†	353 (16.1%)	160 (14.5%)	193 (17.8%)	
Housewife	190 (8.7%)	0 (0%)	190 (17.5%)	<0.0001
Marital status				
Never married	340 (15.5%)	229 (20.7%)	111 (10.2%)	
Not currently married	505 (23.1%)	210 (19.0%)	295 (27.2%)	
Married monogamous	923 (42.1%)	511 (46.2%)	412 (38.0%)	
Married polygamous	423 (19.3%)	156 (14.1%)	267 (24.6%)	<0.0001
Duration in community (years)				
Less than 1	394 (17.9%)	171 (15.5%)	223 (20.5%)	
1–4	823 (37.6%)	398 (36.0%)	425 (39.2%)	
5–10	668 (30.5%)	351 (31.7%)	318 (29.3%)	
More than 10	305 (13.9%)	186 (16.8%)	119 (11.0%)	<0.0001
Alcohol in past 3 months				
Yes	1160 (52.9%)	652 (56.2%)	508 (43.8%)	
No	1031 (47.1%)	454 (44.0%)	577 (56.0%)	<0.0001
Alcohol before sex in past 3 months				
Yes	944 (43.4%)	470 (49.8%)	474 (50.2%)	
No	1229 (56.6%)	628 (51.1%)	601 (48.9%)	0.545
Use of marijuana				
Yes	302 (13.8%)	241 (21.8%)	61 (5.6%)	
No	1888 (86.2%)	864 (78.2%)	1024 (94.4%)	<0.0001
Condom use in past 12 months				
Yes	806 (40.1%)	535 (52.4%)	271 (27.5%)	
No	1202 (59.9%)	486 (47.6%)	716 (72.5%)	<0.0001
Consistent condom use				
Inconsistent	608 (75.4%)	398 (74.4%)	210 (77.5%)	
Consistent	198 (24.6%)	137 (25.6%)	61 (22.5%)	0.334

* 3 missing education

¶ Seventh day advent/traditionist

† construction/mechanic/government/clerical.

## HIV prevalence and associated factors

Overall, the HIV prevalence was 26.7% (95% CI: 24.8, 28.6%) and was significantly higher among women than men (32.6% vs. 20.8%, *p*<0.0001) (Table 2). HIV prevalence increased with age from 18.3% in 18–24 year-olds, 27.4% in 25–29 year-olds, 31.5% in 30–39 year-olds and 30.8% among those aged 40–49 years. However, the prevalence decreased with increasing levels of education – 39.8% among those with no formal education, 28.6% for primary-level education and 19.8% for post-primary education. High HIV prevalence was observed among participants in polygamous marriages and those previously married but not married at the time of the survey, those who reported alcohol consumption in past three months, alcohol use before sex and those who reported use of illicit drugs such as marijuana. People in FFC are involved in multiple occupations and it is noteworthy that the HIV prevalence was lowest among persons directly doing fishing and fishing-related activities (22.4%), but was higher among stay-at-home housewives (32.1%), farmers (33.1%), and highest among those owning or working in a bar/lodge/restaurant business (37%). The latter group includes commercial sex workers, which may explain the high HIV prevalence. The HIV prevalence did not differ by tribe/ethnicity (*p*=0.49), condom use in the past 12 months (*p*=0.15), or duration of stay in community (*p*=0.54). Overall, the mean (SD) age at sexual debut was significantly lower among HIV positives [16.3 (5.5) years] than negatives [17.6 (9.1) years], *p*=0.0001. The same trend was observed when stratified by gender; among men 17.2 (2.9) years in positives versus 18.2 (9.3) in negatives, *p*=0.004 and among women 15.8 (6.6) years in positives compared to 16.8 (8.9) years in negatives, *p*=0.03 (not shown).

**Table 2:** HIV prevalence, unadjusted and adjusted prevalence risk ratios (PRRs) of factors associated with HIV-1 prevalence in fishing communities in Uganda

	HIV-positive	Unadjusted PRRs	Adjusted PRRs	
		
Characteristic	% (*n*/*N*)	(95% CI)	(95% CI)	*p*
All				
Positive	26.7% (584/2191)			
Negative	73.3% (1,607/2191)			
Age (years)				
18–24	18.3% (113/616)	1 (ref)	1 (ref)	
25–29	27.4% (155/566)	1.49 (1.17, 1.90)	1.28 (0.99, 1.64)	0.06†
30–39	31.5% (231/733)	1.72 (1.37, 2.15)	1.40 (1.10, 1.79)	0.007††
40–49	30.8% (85/276)	1.68 (1.27, 2.22)	1.41 (1.04, 1.92)	0.025††
Sex				
Male	20.8% (230/1106)	1 (ref)	1 (ref)	
Female	32.6% (354/1085)	1.57 (1.36, 1.81)	1.50 (1.20, 1.87)	<0.0001††
Highest education level*				
Post-primary	19.8% (140/708)	1 (ref)	1 (ref)	
Primary	28.6% (370/1294)	1.45 (1.19, 1.76)	1.24 (1.02, 1.52)	0.033††
None	39.8% (74/186)	2.01 (1.52, 2.67)	1.60 (1.20, 2.14)	0.001††
Religion				
Pentecostal	19.8% (39/197)	1 (ref)	1 (ref)	
Roman Catholic	30.2% (269/890)	1.53 (1.09, 2.14)	1.27 (0.89, 1.80)	0.184
Protestant/Anglican	25.7% (154/600)	1.30 (0.91, 1.84)	1.11 (0.77, 1.60)	0.564
Muslim	22.3% (94/421)	1.13 (0.78, 1.64)	1.03 (0.71, 1.51)	0.871
Others	33.7% (28/83)	1.70 (1.05, 2.77)	1.46 (0.89, 2.39)	0.138
Occupation				
Fishing/fishing-related	22.4% (233/1038)	1 (ref)	1 (ref)	
Trade/business	26.0% (58/223)	1.16 (0.87, 1.54)	1.04 (0.76, 1.43)	0.798
Bar/lodge/restaurant	37.0% (95/257)	1.65 (1.30, 2.09)	1.19 (0.90, 1.58)	0.217
Farming	33.1% (43/130)	1.47 (1.06, 2.04)	1.23 (0.88, 1.74)	0.223
Housewife	32.1% (61/190)	1.43 (1.08, 1.90)	1.16 (0.83, 1.62)	0.370
Others	26.6% (94/353)	1.19 (0.93, 1.51)	1.13 (0.88, 1.45)	0.345
Marital status				
Never married	10.3% (35/340)	1 (ref)	1 (ref)	
Not currently married*	34.6% (175/505)	3.37 (2.34, 4.84)	2.20 (1.50, 3.24)	<0.0001††
Married monogamous	25.2% (233/923)	2.45 (1.72, 3.50)	1.86 (1.28, 2.71)	0.001††
Married polygamous	33.3% (141/423)	3.24 (2.24, 4.69)	2.20 (1.48, 3.28)	<0.0001††
Alcohol in past 3 months				
No	20.8% (214/1030)	1 (ref)	1 (ref)	
Yes	31.9% (370/1160)	1.31 (1.17, 1.46)	1.19 (0.98, 1.43)	0.07†
Alcohol before sex in past 3 months				
No	21.3% (262/1229)	1 (ref)	1 (ref)	
Yes	33.9% (320/944)	1.59 (1.35, 1.87)	1.27 (1.06, 1.53)	0.01††
Use of marijuana				
No	25.6% (483/1888)	1 (ref)	1 (ref)	
Yes	33.4% (101/302)	1.31 (1.09, 1.56)	1.40 (1.11, 1.76)	0.004††

†† Statistically significant at *p*<0.05

† borderline significant at *p*<0.05

* previously married but not at the time of survey.

At unadjusted analysis, statistically significant predictors of HIV prevalence in the fishing communities included age (>25 years), female gender, no formal education or primary education, religion, working in a bar/lodge/restaurant, previous and current marriage, alcohol consumption in the past three months, alcohol use before sex and the use of marijuana (Table 2). However, acquiring a new sexual partner in 3 or 12 months prior to the survey was not associated with high HIV-1 prevalence; relative to those who reported no new sexual partners, HIV prevalence was neither associated with having at least one new sex partner in the past 12 months (*p*=0.93) or in the past 3 months (*p*=0.55) nor with having higher numbers of new partners in the past three months (*p*=0.51).

After adjustment, factors that remained statistically significantly associated with HIV prevalence were age (>25 years), female gender, no formal education or primary education, previous and current marriage, alcohol use before sex and the use of marijuana. Religion, occupation and alcohol consumption in the past three months lost statistical significance after multivariable analyses.

The adjusted HIV adj.PPR was higher among women compared to men (adj.PPR=1.50, 95%; 1.20, 1.87) and among participants aged 30–39 years (adj.PPR=1.40, 95%; 1.10, 1.79) and 40–49 years (adj.PPR=1.41, 95%; 1.04, 1.92) compared to their counterparts aged 18–24 years. Relative to the never married, the adjusted HIV prevalence was 2.2 times higher (adj.PPR=2.20, 95% CI: 1.50, 3.24) for previously married but not married at the time of the survey, 1.86 higher (adj.PPR=1.86, 95% CI: 1.28, 2.71) among those monogamously married and 2.2 times higher among the polygamous married (adj.PPR=2.20, 95% CI: 1.48, 3.28).

No formal education at all and primary-level education were both associated with higher HIV prevalence than those with post-primary education (adj.PPR=1.60, 95% CI: 1.20, 2.14 and adj.PPR=1.24, 95% CI: 1.02, 1.52, respectively). Other factors associated with HIV prevalence were alcohol use before sex (adj.PPR=1.27, 95% CI: 1.06, 1.53) and the use of marijuana (adj.PPR=1.40, 95% CI: 1.11, 1.76).

## WTP in future HIV vaccine trials

Knowledge about HIV vaccine testing and WTP in future vaccine trials were assessed using hypothetical questions since there was no actual vaccine trial going on in the study population. As shown in Table 3, 53.7% (1176/2191) were aware of existing efforts to develop a HIV preventive vaccine and the awareness was higher in men than women (58% vs. 49.2%, *p*<0.0001). The major source of information on HIV vaccine development in this population was mass media (radio and newspapers), which accounted for 55.2%, followed by HIV research organizations (19.1%). We wanted to establish if participants distinguished between participating in a HIV vaccine trial in general terms such as being supportive versus participating as a trial volunteer. Although 94.5% indicated WTP in future HIV vaccine trials in general, when it came to participating as a trial volunteer, WTP reduced to 89.3% (1953/2191). WTP was significantly higher in men than women (91.2% vs. 87.3%, *p*=0.004) and among island communities compared to lakeshore ones (90.4% vs. 85.8%, *p*=0.004) (not shown). WTP as a trial volunteer did not differ by age group, education status, occupation, marital status and HIV status at baseline (not shown). The major concerns of those not willing to participate included a fear of vaccine side effects 43.2% (80/185) and the potential for the experimental vaccine to cause HIV/AIDS 27% (50/185).

**Table 3:** Awareness of HIV vaccine development and willingness to participate in future HIV vaccine studies (WTP) by gender (*n*=2191)

	All	Male	Female	
		
	2191	1106 (50.5%)	1085 (49.5%)	*p*
Awareness of HIV vaccine development				
Yes	1176 (53.7%)	642 (58.0%)	534 (49.2%)	
No	1015 (46.3%)	464 (41.5%)	551 (50.8%)	<0.0001
Source of information on HIV vaccines				
HIV research organizations	226 (19.1%)	116 (18.0%)	110 (20.5%)	
Hospitals/clinics	160 (13.5%)	65 (10.1%)	95 (17.7%)	
Radio/newspapers	652 (55.2%)	392 (60.8%)	260 (48.4%)	
Other sources*	143 (12.1%)	71 (11.0%)	72 (13.4%)	<0.0001
Willingness to participate (WTP) in HIV vaccine trial in any way				
Yes	2077 (94.8%)	1065 (96.3%)	41 (3.7%)	
No	114 (5.2%)	1012 (93.3%)	73 (6.7%)	0.001
WTP in HIV vaccine trial as a trial participant				
Willing	1953 (89.3%)	1007 (91.2%)	946 (87.3%)	
Not willing	187 (8.5%)	82 (7.4%)	105 (9.7%)	
Not sure	48 (2.2%)	15 (1.4%)	33 (3.0%)	0.004

* Mainly community meetings and friends.

## Discussion

In a general population of fisher folk from eight communities in three Uganda districts of Mukono, Wakiso and Kalangala around Lake Victoria, we found a HIV-1 prevalence of 26.7% and WTP in hypothetical HIV vaccine trials of 89.3%. The adjusted HIV prevalence proportion ratio was higher among women, persons aged 30 years or more, those in polygamous marital relationships, with lower education status, and those who used alcohol and marijuana before sex. However, being a fisherman per se was not a significant predictor of HIV prevalence in this study. Although it is believed that the high HIV rates in fishing communities results from mobile and risk-taking nature of fishermen, we found a lower HIV prevalence among fishermen than housewives, farmers and those who owned or worked in a bar/lodge/restaurant. It is not clear to us why individuals directly involved in fishing had a lower prevalence of HIV in this study. More qualitative and quantitative studies are needed to elucidate the risk profiles of different subgroups of individuals in fishing communities.

The above findings show that HIV prevalence in the general fisher-folk population is similar to that observed in the “high-risk” fisher-folk individuals (26.7% and 28.8%, respectively) [4] and in other fishing communities around Lake Albert in Uganda (24%) and in Kisumu, Kenya (26%) [8, 9]. These findings provide more evidence that within a mature and generalized HIV epidemic in Uganda, fishing communities around Lake Victoria have a higher HIV burden than the general population – about four times higher prevalence than the national average [3, 5]. This is not very surprising given that first cases of HIV in the country were identified from a lakeshore fishing community in Rakai district, south western Uganda [10] and that these communities tend to be socially marginalized, and have high levels of risky behaviours and sexually transmitted infections [2–4, 8, 11] . In the East and Southern African region, which is most heavily burdened by the HIV epidemic, there is a large fisher-folk population. In Uganda alone, there are approximately two and a half million people engaged in fishing activities, and fishing contributes over 6% to the national gross domestic product [3].

We also found a high level of WTP (89.3%) which to our knowledge is one of the highest reported in a general population study in sub-Saharan Africa. With the exception of a community-based study in Masaka, Uganda where WTP was 95% [12], most previous studies in Africa found levels of WTP ranging between 40 and 77% [13–15]. In this study, WTP was significantly higher among men than women and this observation differed from a previous Ugandan study in which WTP did not vary by gender [15]. The main reasons for lack of WTP were fear of vaccine side effects (43.2%) and the potential for the experimental vaccine to cause HIV/AIDS 27%. It was not clear whether the latter referred to a candidate vaccine actually causing HIV infection or to vaccine-induced seropositivity (VISP). Levels of VISP of 42% have been previously reported in multicentre study with participants from Africa although not particularly fishing communities [16]. Studies in India have reported stigma and perception of not being at risk of HIV as reasons for lack of WTP [17, 18]. If fishing communities are to be involved in preventive HIV vaccine trials, issues such as vaccine side effects, the fear of an experimental vaccine causing HIV/AIDS and VISP should be addressed. In order to understand WTP better and elucidate its determinants FFC, more studies focusing on stigma, preventive misconception, optimism, self-efficacy, altruism and a thorough understanding of what is expected of and required from HIV vaccine trial participants, are urgently needed. A previous study in Uganda found that delayed pregnancy (for women), larger blood draws and the possibility of receiving either candidate vaccine or placebo through randomization significantly reduced WTP from 95% to 23%, and 55% and 73%, respectively [12].

We want to emphasize two points regarding WTP. First, although participants’ WTP is essential for the enrolment in trials, high levels of pre-trial hypothetical WTP may not necessarily imply actual trial participation. A US study that compared hypothetical and actual WTP found that only 20% of the persons that stated hypothetical WTP actually enrolled in the subsequent vaccine trial [19]. Second, in this study WTP was assessed in a hypothetical manner since there was no actual vaccine trial enrolment in these communities at the time of the study hence the reported findings on WTP should be interpreted accordingly.

The strengths of this study include the community representative study population, a large sample size and the wide geographical representation (study communities had not been involved in previous HIV epidemiological studies and came from three Ugandan districts). Nonetheless, this study had some limitations. First, we did not assess the magnitude of sexually transmitted infections and their association with HIV. However, we believe that this has been extensively documented in previous studies that probably not much new knowledge would have been added [20–22]. Second, we did not quantitatively assess the levels of circumcision and willingness to receive medical male circumcision but a qualitative study in the same communities indicated low levels of circumcision with very high demand for the service (pc Simon Sigirenda, Social Sciences Coordinator, UVRI-IAVI HIV Vaccine Program). Third, the study included persons aged 18–49 years and as such we could not determine WTP among adolescents, yet previous studies have reported high WTP levels in adolescents [15, 23]. Fourth, the study was conducted among persons aged 18–49 years excluding adolescents (11–17 years), which is another high-risk population group that would be ideal for HIV vaccine trails.

## Conclusions

This study adds to more emerging evidence that in addition to commercial sex workers [24, 25], fishing communities in Uganda are high HIV burdened groups with the prevalence in the general fisher-folk population being similar to that observed in the “high-risk” individuals.
